# Marking Vertices to Find Graph Isomorphism Mapping Based on Continuous-Time Quantum Walk

**DOI:** 10.3390/e20080586

**Published:** 2018-08-08

**Authors:** Xin Wang, Yi Zhang, Kai Lu, Xiaoping Wang, Kai Liu

**Affiliations:** 1 College of Computer Science, National University of Defense Technology, Changsha 410073, China; 2 Science and Technology on Parallel and Distributed Processing Laboratory, National University of Defense Technology, Changsha 410073, China

**Keywords:** graph isomorphism, isomorphism mapping, continuous-time quantum walk, graph mining, data mining

## Abstract

The isomorphism problem involves judging whether two graphs are topologically the same and producing structure-preserving isomorphism mapping. It is widely used in various areas. Diverse algorithms have been proposed to solve this problem in polynomial time, with the help of quantum walks. Some of these algorithms, however, fail to find the isomorphism mapping. Moreover, most algorithms have very limited performance on regular graphs which are generally difficult to deal with due to their symmetry. We propose IsoMarking to discover an isomorphism mapping effectively, based on the quantum walk which is sensitive to topological structures. Firstly, IsoMarking marks vertices so that it can reduce the harmful influence of symmetry. Secondly, IsoMarking can ascertain whether the current candidate bijection is consistent with existing bijections and eventually obtains qualified mapping. Thirdly, our experiments on 1585 pairs of graphs demonstrate that our algorithm performs significantly better on both ordinary graphs and regular graphs.

## 1. Introduction

The problem of graph matching is finding similarities between graphs [1,2]. Exact graph matching is usually known as the graph isomorphism problem, which is judging whether two graphs are topologically the same [3]. The key to graph isomorphism is finding a structure-preserving mapping called isomorphism mapping [4].

**Definition 1** (Isomorphism mapping). *Given two graphs $G_{1}=\left(V_{1},E_{1}\right)$ and $G_{2}=\left(V_{2},E_{2}\right)$, an isomorphism mapping is a bijective mapping between the vertex sets of $G_{1}$ and $G_{2}$*

(1) $$f:V_{1}\to V_{2},$$

*such that for any two nodes ($v_{i},v_{j}\in V_{1}$), they are adjacent in $G_{1}$ if and only if two nodes ($f\left(v_{i}\right),f\left(v_{j}\right)\in V_{2}$) are adjacent in $G_{2}$.*

Based on Definition 1, we can see that the isomorphism mapping (*f*) is a set of many unit bijections, such as $v_{i}\to f\left(v_{i}\right)$ and $v_{j}\to f\left(v_{j}\right)$. To make things clear, we use isomorphismmapping or mapping to represent the whole mapping (*f*) and use unitbijection or bijection to represent a particular bijective relation between two nodes. Based on unit bijections, we can establish an isomorphism mapping and ascertain whether two graphs are isomorphic. Accordingly, the solution to the graph isomorphism problem often provides an isomorphism mapping rather than a judgement.

**Definition 2** (Isomorphic). *Two graphs $G_{1}=\left(V_{1},E_{1}\right)$ and $G_{2}=\left(V_{2},E_{2}\right)$ are isomorphic if there is an isomorphism mapping (f) between the vertex sets of $G_{1}$ and $G_{2}$.*

Graph matching and graph isomorphism are widely used in various areas, including biochemistry [5], chemical database searches [6], electronic design automation [7], information retrieval [8], pattern recognition [9], and many other areas [10]. Because of its applications, graph isomorphism has been deeply studied by scholars and researchers. It is considered to be NP (nondeterministic polynomial-time) in computational complexity theory, but it is unknown whether it is NP-complete or not [11].

Some of the most influential isomorphism algorithms, including VF2 (an improved graph-matching algorithm proposed by Vento) [12], VF3 (a novel matching algorithm proposed by Vento) [13,14], RI (a matching algorithm proposed by Bonnici) [5], and LAD (Local All Different proposed by Solnon) [15], are based on the tree search. Starting with an empty mapping, they add verified unit bijections one after another and eventually obtain an isomorphism mapping. They often utilize backtracking and heuristics for pruning. Hence, they are among the most effective classical algorithms. Moreover, they can always give a correct isomorphism mapping, because they utilize verification while exploring. Although these algorithms are perfectly accurate, they are computationally complex. Their complexity may become exponential in the worst cases. In fact, most classical algorithms for graph isomorphisms have a high computational complexity, and thus, their performance is usually limited. There are also some other algorithms. For instance, Gori announced a method based on random walks [16]. It solves the problem in polynomial time, but only works on certain kinds of graphs. In 2015, Babai proposed a quasi-polynomial time algorithm [17], but the complexity of it was still not very satisfying.

Quantum walks gave people new inspiration to design algorithms. These algorithms can work on all kinds of graphs in polynomial time. Although they lack theoretical proof, their experimental accuracy is satisfying. Based on either the continuous-time quantum walk (CTQW) [18] or the discrete-time quantum walk (DTQW) [19], diverse algorithms have been proposed to solve the isomorphism problem with high accuracy and polynomial complexity. Douglas and Wang proposed an algorithm to distinguish two graphs using DTQW [20]. It is relatively accurate, with a complexity of $O(|V{|}^{7})$. David Emms’ algorithm, called Emms, is based on CTQW [21], and he gives a discrete-time version likewise [22]. The two algorithms have both polynomial time complexity, but they have very limited performance on regular graphs. Regular graphs are usually highly symmetric because each vertex has the same degree. Algorithms often find them difficult to distinguish, and thus produce incorrect isomorphism mappings.

The algorithm named Qiang1 proposed by Qiang utilizes CTQW to ascertain whether two graphs are isomorphic [23]. It is accurate and fast, with a complexity of $O(|V{|}^{5})$ [23]. Nevertheless, it can only provide a judgement rather than a detailed isomorphism mapping. Moreover, its performance is also limited when faced with regular graphs. Its extension, the Intuitive Method, tries different unit bijections and integrates them into an isomorphism mapping [24]. It is effective but still suffers from poor accuracy on regular graphs. Another algorithm by Qiang, named Qiang3 [24], can both ascertain the isomorphism between two graphs and obtain an isomorphism mapping. Although Qiang3 claims to achieve improved accuracy, especially on regular graphs, its performance is still not very satisfying.

In a word, most algorithms based on quantum walks are still far from satisfactory. Some cannot provide detailed isomorphism mapping. Moreover, most algorithms have limited performance on regular graphs and thus output incorrect isomorphism mappings.

In this paper, we propose IsoMarking, inspired by Qiang’s algorithms. IsoMarking can discover the detailed isomorphism mapping between two graphs. It not only reduces the symmetry by marking vertices but also keeps the unit bijections qualified for a correct isomorphism mapping. It achieves better accuracy and, in particular, performs better on regular graphs. Our main contributions are listed as follows:

1. We propose the idea of using vertex markings to reduce the impact of symmetry provided by regular graphs.
2. We design a delicate and practical mechanism to mark diverse vertices and implement a detailed algorithm.
3. We propose an algorithm to ascertain whether the current unit bijection is consistent with the existing ones so that the isomorphism mapping is correct.
4. We conduct experiments on 1585 pairs of graphs and discover that IsoMarking performs significantly better on both ordinary graphs and regular graphs.

The rest of paper is organized as follows. Section 2 introduces related works, including quantum walks and isomorphism algorithms based on quantum walks. Section 3 introduces IsoMarking. Firstly, we discuss the impact of symmetry that regular graphs have. Then, we introduce the basic idea of marking vertices to reduce the symmetry. Based on a delicate marking mechanism, we present the detailed algorithm and a further discussion in Section 3. In Section 4, we take a pair of pentagrams as an example and see how IsoMarking works out correct isomorphism mapping between regular graphs compared with the Intuitive Method. Experiments are conducted in Section 5 to evaluate IsoMarking and other algorithms. The paper is concluded in Section 6.

## 2. Related Works

This section discusses related works. Firstly, we introduce some information concerning quantum walks. Then, we discuss isomorphism algorithms based on quantum walks in detail.

### 2.1. Quantum Walks

The quantum walk is the quantum analogue of classical random walks [25]. It introduces several kinds of quantum states to represent the current walker. The walker is a superposition described by a probability distribution over basis quantum states. Analogous to the classical random walk, the quantum walk can be described by different models, including the continuous-time quantum walk (CTQW) [18] and the discrete-time quantum walk (DTQW) [19]. A CTQW introduces position states to describe the current position of walker and utilizes the continuous unitary transformation to control the state evolution. In graph G=(V,E), each position, namely, each node (v∈V), is represented by a column vector (|v〉), which is the basis state of the quantum walk in graph *G*. The walker is in a superposition of basis states, and each basis state has a corresponding probability amplitude ($\alpha_{v}\left(t\right)$). The probability amplitude is a complex number, and the square of its modulus equals the probability of basis state |v〉, namely the probability for the walker to be at node *v*. Consequently, the state of CTQW $|\varphi_{t}\rangle$ at time *t* can be written as follows:

(2) $$|\varphi_{t}\rangle =\sum_{v\in V}\alpha_{v}\left(t\right)|v\rangle$$

(3) $$\sum_{v\in V}{|\alpha_{v}\left(t\right)|}^{2}=1.$$

Given an initial state vector $|\varphi_{0}\rangle$, we can compute the state vector with Equation (4). We usually choose the adjacency matrix or the Laplacian matrix as the Hamiltonian matrix (*H*):

(4) $$|\varphi_{t}\rangle =e^{-iHt}|\varphi_{0}\rangle .$$

DTQW, in contrast, introduces position states and coin states to describe the current position and the moving direction, respectively. It is assumed that the walker is on an integer axis, and that the position state at point n∈Z can be represented by a basis state (|n〉). The moving direction can be described by two basis coin states |↑〉 and |↓〉 which represent goingright and goingleft, respectively. At position *n*, the walker is in a superposition of |n〉⊗|↑〉 and |n〉⊗|↓〉. Each step of DTQW is a unitary transformation that has two operations on the current state. The first operation is called the coin flip transformation which changes the state of moving directions. The second is the shift operation, and it changes the position states. If the initial state and transformations are given, the quantum walk can be performed. Similarly, the probability of a basis state comes from the probability amplitude. On graphs, DTQW is similar, although the walk is more complicated.

Quantum walks can contribute to exponential speedup and are widely used in many algorithms. They can be applied to many fast search algorithms [26,27,28]. Some simulated annealing algorithms are also based on quantum walks [29]. Likewise, quantum walks can be used in mathematics, including graph theory [30] and the element distinctness problem [31].

### 2.2. Isomorphism Algorithms Based on Quantum Walks

Quantum walks are very sensitive to topological structures. Therefore, the probability amplitude can reveal topological patterns and roles. Consequently, quantum walks are widely used to solve many problems, including the computation of graph similarity [32] and graph kernels [33] and the detection of symmetries [34]. Likewise, quantum walks are applied in isomorphism algorithms to promote their performance. Moreover, quantum walks make it possible to solve the problem in polynomial time.

In 2008, Douglas and Wang [20] proposed the Douglas method based on DTQW, with a complexity of $O(|V{|}^{7})$. After performing DTQW, it compares the probability amplitude sets of two graphs. Although the correctness of Douglas is not theoretically proven, experiments demonstrate that it can distinguish most non-isomorphic graphs. In fact, for most isomorphism algorithms based on quantum walks, the correctness comes from massive experiments, rather than a theoretical proof.

Likewise, David Emms proposed two algorithms [21,22] based on CTQW and DTQW, respectively. Although these two algorithms utilize different quantum walks, they have similar mechanisms. Firstly, they co-join two graphs by a layer of indicator vertices and construct an auxiliary graph. Then, two quantum walks are simulated on two graphs in parallel. The probability amplitude of indicator vertices can reflect the quantum interference between two quantum walks. The interference amplitudes between equivalent nodes in two graphs are usually close to 0; therefore, such equivalent nodes can be detected. Emms establishes unit bijections according to equivalent nodes and then obtains the isomorphism mapping. Both algorithms are polynomial-time. However, they often produce incorrect mapping between regular graphs; hence, they have very limited performance on regular graphs. Regular graphs are highly symmetric; therefore, Emms’s auxiliary graph is even more symmetric. Both algorithms only consider the global structure rather than local patterns. Thus they can be confused by such equivalence between graphs.

Inspired by Douglas and Wang, Qiang proposed an enhanced approach called Qiang1 [23]. After performing CTQW on both graphs, it analyses the result and ascertains whether two graphs are isomorphic or not. Qiang1 focuses on pairs of vertices with an equal probability amplitude at some time, which can reflect the similarity between local structures. Qiang1 introduces the generalized similar vertex set and studies the similarity between graphs according to the probability amplitudes. Besides, it adds self-loops to reveal local topological information. Qiang’s algorithm is accurate and fast. Its complexity is $O(|V{|}^{5})$ when the size of generalized similar vertex set is 2, and the complexity drops to $O(|V{|}^{4})$ when the size is 1. Nevertheless, it can only provide a judgement rather than an isomorphism mapping. Moreover, it may distinguish regular graphs incorrectly, and therefore, its performance on regular graphs is limited.

Qiang extended the algorithm and proposed the Intuitive Method. This method can find an isomorphism mapping between isomorphic graphs by calling Qiang1 repeatedly [24]. At each turn, the algorithm randomly chooses one node from each graph and tries to establish a unit bijection between them. For node $v_{1}$ in graph $G_{1}$ and node $v_{2}$ in $G_{2}$, the algorithm adds two new nodes only connected to $v_{1}$ and $v_{2}$, respectively. It generates two new graphs, where the only changed parts are the local structures of $v_{1}$ and $v_{2}$. If $v_{1}$ and $v_{2}$ are equivalent, the topological changes are the same. With the original isomorphic graphs, the new graphs are also isomorphic. Then, the algorithm establishes a unit bijection between $v_{1}$ and $v_{2}$, denoted as $v_{1}\to v_{2}$. If the two nodes are not equivalent, the probability amplitudes will be significantly different, because the quantum walk is extremely sensitive to topological changes. Accordingly, the new graphs are not isomorphic, and the candidate unit bijection $v_{1}\to v_{2}$ fails. With repeated trials, unit bijections between nodes can be established one by one, and the whole isomorphism mapping is obtained eventually. This strategy works effectively on ordinary graphs, but it performs poorly on regular graphs. There are too many possible bijections because regular graphs are highly symmetric. Any unit bijection alone is suitable, but unit bijections can have conflict with each other. A simple integration of unit bijections can be problematic. Thus, the Intuitive Method often produces incorrect mappings.

Qiang further proposed another algorithm, Qiang3 [24], which can both ascertain the isomorphisms between graphs and find isomorphism mappings. It studies the differences between the probability amplitude sets of two graphs. If two probability amplitudes are the same, the corresponding nodes can establish a unit bijection. It establishes an isomorphism mapping based on such bijections. If the algorithm is not able to find an isomorphism mapping, then the two graphs are non-isomorphic. Likewise, Qiang3 adds self-loops to vertices in order to reflect the local topological information, which can reduce the impact of symmetry to some extent. Qiang3 is $O(|V{|}^{4})$ and is claimed to achieve improved performance on regular graphs, although the accuracy is still not satisfying.

In this paper, we propose an algorithm based on Qiang’s algorithms. It can not only find the isomorphism mapping between graphs but also performs better on regular graphs.

## 3. IsoMarking

This section introduces IsoMarking. Firstly, we discuss the impact of symmetry to see why regular graphs are difficult for isomorphism algorithms. Then, we introduce the basic idea of marking vertices to reduce the symmetry. In the third part, we design a delicate mechanism to mark vertices, followed by a detailed algorithm. In addition, we present a further discussion concerning IsoMarking, including the computational complexity.

### 3.1. The Impact of Symmetry

Although the quantum walk is sensitive to topological structures, its performance suffers from the presence of symmetrical structures. Both DTQW and CTWQ rely on the probability amplitude to reveal the topological equivalence between graphs, based on which algorithms can establish unit bijections. Symmetric graphs, however, usually have many equivalent vertices that are confusing.

In regular graphs, nodes have the same number of neighbours. Therefore, regular graphs are highly symmetric and even automorphic which means the graph can be mapped onto itself by a structure-preserving permutation mapping [35]. Such strong symmetry can result in too many equivalent nodes and candidate unit bijections. Any candidate unit bijection alone is suitable because it comes from some equivalence. More unit bijections, however, can conflict with each other, because their integration as a mapping is not always structure-preserving. As a consequence, the mapping does not always qualify for an isomorphism mapping.

**Figure 1:**
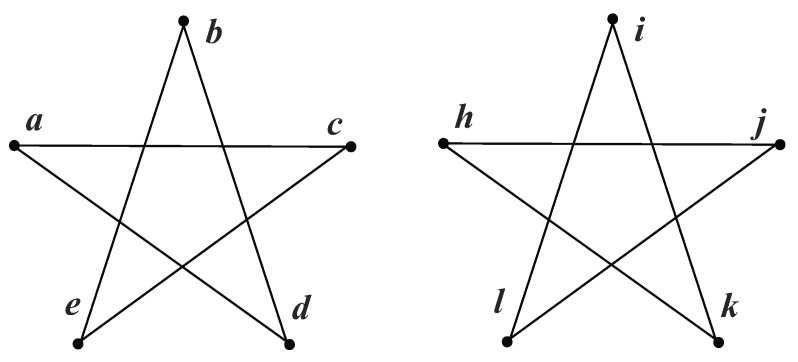
Two pentagrams.

For instance, each pentagram in Figure 1, has five equivalent nodes. When we perform a quantum walk on both sides, the probability amplitudes are always the same, as shown in Table 1 and Table 2. Consequently, any two nodes are equivalent. One node in a pentagram can establish a unit bijection with any node in another pentagram. Hence, there are 25 candidate unit bijections in total. Then we choose five unit bijections, under the condition that every node is used only once. However, such bijections still cannot make an isomorphism mapping. For instance, the output can be {a→i,b→l,c→h,d→j,e→k}. That result is not structure-preserving, because *i* and *l* are adjacent, while *a* and *b* are not.

Unfortunately, most algorithms treat graphs globally, and thus they make little effort to reduce the symmetry. Therefore, most algorithms perform poorly on regular graphs. They usually find it difficult to distinguish between regular graphs. Even though the isomorphism is correctly judged, the produced isomorphism mapping is still problematic.

### 3.2. Marking Vertices

The idea for coping with regular graphs and other symmetric graphs is to reduce the symmetry. Inspired by the Intuitive Method, we can add new adjacent nodes to vertices, which we call markingvertices. Different from the Intuitive Method, IsoMarking marks vertices not only to try diverse unit bijections but also to reduce the symmetry.

It is intuitive to reduce the symmetry by marking vertices. Since a regular graph is highly symmetric, there are several equivalent parts. If one part is marked by new neighbours while other parts are marked differently or not marked, these parts are no longer equivalent. Therefore, the symmetry is reduced. In addition, the quantum walk is so sensitive that only a few changes can give rise to dramatically different probability amplitudes. Therefore the influence of marking vertices on symmetry can be easily detected.

IsoMarking produces a more complicated mechanism to generate a new graph. We introduce four types of marking operations to mark more node pairs at the same time. Suppose we aim to mark node $v_{1}$ in graph $G_{1}=\left(V_{1},E_{1}\right)$ and $v_{2}$ in $G_{2}=\left(V_{2},E_{2}\right)$. Because marking is operated equivalently on both sides, we only describe the operation of each marking type on $G_{1}$:

1. Add one node $v_{|V_{1}|+1}$ to $G_{1}$ which is only adjacent to $v_{1}$;
2. Add two nodes $v_{|V_{1}|+2},v_{|V_{1}|+3}$ to $G_{1}$ which are only adjacent to $v_{1}$;
3. Add two nodes $v_{|V_{1}|+4},v_{|V_{1}|+5}$ to $G_{1}$ which are adjacent to $v_{1}$ and to each other;
4. Add three nodes $v_{|V_{1}|+6},v_{|V_{1}|+7},v_{|V_{1}|+8}$ to $G_{1}$ which are adjacent to $v_{1}$ and to one another.

The function of marking vertices is implemented in Algorithm 1. Given a graph, this function can mark, at most, four nodes using different marking types and then a new graph is returned. In Lines 3–6, node $v_{a}$ is marked by the first marking type, and in Line 7–10 i$v_{b}$ is marked by the second type. Similarly, $v_{c}$ and $v_{d}$ are marked by the third and fourth types, respectively. Vertices are marked only if they are in the graph, and thus, we can input illegal node (−1) to indicate no more marking. The computational complexity of Mark() is O(1) because each line of Algorithm 1 is O(1). Since the marking is operated equivalently on two graphs, Algorithm 1 is supposed to be called twice.

Note that we present four marking types in our paper. In fact, the number of marking types is not fixed. Intuitively, the number of marking types is related to the performance. There are some regular graphs requiring more marking types, while less marking types are sufficient sometimes. In Section 4, although IsoMarking uses four marking types, we can see that a pentagram marked by three types is no longer symmetric. Our algorithms are open to different numbers of marking types. We can easily modify our algorithms for a different number of types.

**Algorithm 1** Mark
1:**Input: **G=(V,E), nodes to mark $v_{a},v_{b},v_{c},v_{d}$;2:$V_{new}=V,E_{new}=E$;3:
**if** $v_{a}\in V$ **then**
4:    $V_{new}=V_{new}\cup \left\{v_{|V|+1}\right\}$;5:    $E_{new}=E_{new}\cup \left\{\left\{v_{a},v_{|V|+1}\right\}\right\}$;6:
**end if**
7:
**if** $v_{b}\in V$ **then**
8:    $V_{new}=V_{new}\cup \left\{v_{|V|+2},v_{|V|+3}\right\}$;9:    $E_{new}=E_{new}\cup \left\{\left\{v_{b},v_{|V|+2}\right\},\left\{v_{b},v_{|V|+3}\right\}\right\}$;10:
**end if**
11:
**if** $v_{c}\in V$ **then**
12:    $V_{new}=V_{new}\cup \left\{v_{|V|+4},v_{|V|+5}\right\}$;13:    $E_{new}=E_{new}\cup \left\{\left\{v_{c},v_{|V|+4}\right\},\left\{v_{c},v_{|V|+5}\right\},\left\{v_{|V|+4},v_{|V|+5}\right\}\right\}$;14:
**end if**
15:
**if** $v_{d}\in V$ **then**
16:    $V_{new}=V_{new}\cup \left\{v_{|V|+6},v_{|V|+7},v_{|V|+8}\right\}$;17:    $E_{new}=E_{new}\cup \left\{\left\{v_{i},v_{j}\right\}|v_{i},v_{j}\in \left\{v_{d},v_{|V|+6},v_{|V|+7},v_{|V|+8}\right\}\right\}$;18:
**end if**
19:**Output: **the new graph $G_{new}=\left(V_{new},E_{new}\right)$.


### 3.3. Detailed Mechanism

Based on different marking types mentioned above, in this part, IsoMarking can establish unit bijections and reduce the impact of symmetry.

IsoMarking constructs isomorphism mapping based on unit bijections. At each turn, it picks up one node from each graph and tries to establish a unit bijection between picked nodes. In other words, IsoMarking checks whether the current candidate unit bijection is qualified. IsoMarking uses the first marking type to mark picked nodes and uses the other types to mark the other vertices to reduce the symmetry.

For a better understanding of the mechanism, we start with a simple case. Suppose there are two topologically equivalent nodes $v_{i1}$ and $v_{j1}$ in $G_{1}$ as well as two equivalent nodes $v_{i2}$ and $v_{j2}$ in an isomorphic graph $G_{2}$. We aim to reduce equivalence in the same graph while preserving equivalence between graphs. We can simply mark $v_{i1}$ and $v_{i2}$, and then such equivalence in the same graph no longer exists. If there are three equivalent nodes on both sides ($v_{i1},v_{j1},v_{k1}$ in $G_{1}$, and $v_{i2},v_{j2},v_{k2}$ in $G_{2}$), the method is similar. We mark $v_{i1}$ and $v_{i2}$ in one type, mark $v_{j1}$ and $v_{j2}$ differently, and then, the nodes are no longer equivalent. Therefore, for more equivalent vertices, we just need more marking types to distinguish them. We introduce three marking types to reduce the symmetry. More marking types are possible, although three marking types are usually powerful enough. Sometimes they can even change the regular graph into a non-symmetric ordinary graph, as shown in Section 4.

As a result, we can mark equivalent vertices in the same graph to distinguish them and reduce the symmetry. Nevertheless, there are still two problems remaining, as follows:

1. Marking vertices can easily distinguish equivalent nodes in the same graph, but how can we find those equivalent nodes?
2. When we mark nodes in one graph, we are also supposed to mark corresponding nodes equivalently in the other graph. How can we successfully find the corresponding nodes?

For the first problem, we do not bother to find such nodes. Instead, we assume every node may have equivalent nodes in the same graph. We take all vertices into consideration and mark them in some order with their equivalent nodes in the other graph.

The answer to the second problem is crucial. Our aim is to find equivalent nodes between two graphs. Before that, however, we want to find equivalent nodes in the other graph to mark them and reduce the symmetry. There appears to be a circular reasoning. Actually, those equivalent nodes are not supposed to be the same. The key is when we mark vertices to reduce the symmetry, we only mark nodes which are already known to be equivalent. To put it another way, we use the first marking type to mark the nodes of the current candidate bijection and use the other types to mark the existing unit bijections. Given one node in a graph, it is easy to find its equivalent node in the other graph based on the known unit bijections.

From what has been discussed above, we can solve both problems. At each turn, IsoMarking chooses one node from each graph and marks them by the first marking type. Likewise, IsoMarking marks the nodes of the last three established unit bijections. Each pair of nodes is marked by one marking type. Initially, there are not enough existing unit bijections; hence, we just mark as many as possible.

Accordingly, there are usually three existing unit bijections with one candidate unit bijection marked in each new graph. The new graphs are isomorphic if and only if all the four changed parts in one graph are consistent with the corresponding parts in the other graph. The changed structures of existing bijections are already qualified for isomorphism as a whole, guaranteed by the former trial; therefore, they will never disturb the current trial unless the current bijection is not qualified. As long as the changed structure of current candidate bijection can keep two graphs isomorphic, the candidate bijection is acceptable.

The detailed algorithm of IsoMarking is presented in Algorithm 2. It depends on Qiang1() [23] to ascertain whether two graphs are isomorphic. In Line 3, IsoMarking firstly calls Qiang1() to see whether the two graphs are isomorphic, because it makes no sense to try discovering an isomorphism mapping between non-isomorphic graphs. It then tries establishing unit bijections between the nodes from the two graphs. Each time, it studies a pair of nodes and marks them with the last three bijections, as shown in Lines 9–10. If the generated graphs are isomorphic and the current nodes are not used, a new unit bijection is established in Line 13. In Lines 14–16, the last three bijections are updated when a new bijection is accepted. If there are not enough existing bijections, we use −1 to indicate no more marking when calling Mark().

**Algorithm 2** IsoMarking
1:**Input: **$G_{1}=\left(V_{1},E_{1}\right),G_{2}=\left(V_{2},E_{2}\right)$;2:Mapping=∅;3:
**if** $Qiang1(G_{1},G_{2})==False$ **then**
4:    **exit(1)**;5:
**end if**
6:last1=last2=last3=(−1,−1);7:**for** each $v_{i}\in V_{1}$ **do**8:    **for** each $v_{j}\in V_{2}$ **do**9:        $G_{1new}=Mark\left(G_{1},v_{i},last1\left(0\right),last2\left(0\right),last3\left(0\right)\right)$;10:        $G_{2new}=Mark\left(G_{2},v_{j},last1\left(1\right),last2\left(1\right),last3\left(1\right)\right)$;11:        **if** $Qiang1(G_{1new},G_{2new})==True$ **then**12:           **if** $v_{j}$ is not already used in Mapping **then**13:               $Mapping=Mapping\cup \{v_{i}\to v_{j}\}$;14:               last3=last2;15:               last2=last1;16:               $last1=(v_{i},v_{j})$;17:               **break**;18:           **end if**19:        **end if**20:    **end for**21:
**end for**
22:**Output: **the isomorphism mapping Mapping.


### 3.4. Further Discussion

We can understand IsoMarking from another perspective. Most algorithms only establish unit bijections one after another, and simply integrate unit bijections into an isomorphism mapping. They ignore the possibility that unit bijections may conflict with each other. Conflicting unit bijections can make the mapping no longer structure-preserving, and thus, those algorithms often fail. IsoMarking marks a current candidate bijection and several existing bijections at the same time; therefore, it can figure out whether the current candidate bijection is consistent with existing ones. If there are conflicts between them, the overall isomorphism can be destructed; hence, the candidate bijection fails. Otherwise, the new graphs are still isomorphic; hence, the current candidate is consistent with existing bijections. By such methods, IsoMarking can avoid potential conflicts between unit bijections. Only unit bijections consistent with existing ones can be accepted.

By marking the existing unit bijections, IsoMarking can both reduce the symmetry and avoid conflicts between unit bijections. Such a powerful mechanism does not function independently, because it also relies on the quantum walk to capture topological changes sensitively.

IsoMarking has a satisfying computational complexity. Since the isomorphic mapping between non-isomorphic graphs is meaningless, we assume the graphs are isomorphic. Accordingly, two graphs have the same number of vertices, namely $|V_{1}\left|=\right|V_{2}|=N$. In Qiang1(), we set the size of the generalized similar vertex set to be 1, which is accurate enough, so the complexity of Qiang1() in Line 3 drops from $O(N^{5})$ to $O(N^{4})$. There is a nested loop in Lines 9–19. Therefore, the codes are executed for $O\left(N\cdot N\right)=O\left(N^{2}\right)$ times. In Lines 9–10, Mark() is O(1), while Qiang1() in Line 11 is $O({\left(N+8\right)}^{4}=N^{4})$. In fact, IsoMarking adds, at most, eight nodes to each graph; hence, there is little influence on complexity. Line 12 can be simply implemented by an array of Boolean variables within O(1). The rest codes are all O(1). Consequently, the overall complexity is $O\left(1+N^{4}+N^{2}\cdot \left(1+N^{4}\right)\right)=O\left(N^{6}\right)$—the same as the Intuitive Method.

## 4. Case Study

This section compares IsoMarking with another algorithm. We chose the Intuitive Method because it is easy to understand and somewhat similar to IsoMarking. We performed both algorithms on a pair of pentagrams, as shown in Figure 1. The pentagram is a regular graph and is highly symmetric. We aimed to find an isomorphism mapping between two pentagrams.

### 4.1. Results of the Intuitive Method

**Figure 2:**
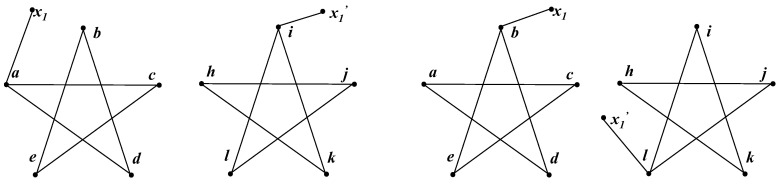
Each step of the Intuitive Method running on pentagrams. (**a**) The algorithm chose *a* and *i*. The generated graphs were the same, and thus a→i was established. (**b**) The algorithm chose *b* and *l*. The generated graphs were the same, and thus b→l was established.

Firstly we show how the Intuitive Method works. At each step, the Intuitive Method randomly picks one node from each graph and tries to establish a unit bijection, as discussed in Section 2.2. We assumed that it picked node *a* in the left and node *i* in the right, and then a new graph was generated, as shown in Figure 2a. Obviously, the new graphs are the same, and thus it a unit bijection (a→i) was established.

At the second step, the Intuitive Method also picks two nodes randomly and tests the isomorphism. We assumed that it chose *b* and *l*; thus it generated the graphs shown in Figure 2b. The graphs in Figure 2b are isomorphic; hence, the algorithm established another unit bijection (b→l). In fact, each step generated the same graph; therefore, the trial was always successful. After the second step, the algorithm established {a→i,b→l}. Nevertheless, those two bijections were already in conflict with each other, because *i* and *l* were adjacent while *a* and *b* were not. Consequently, whatever occurred at the following steps, it definitely failed in this case.

In fact, if we accept unit bijection a→i, only *h* and *j* qualify for a unit bijection together with *b*. The Intuitive Method, however, considers *b* to be equivalent to the other four nodes in the right pentagram. Thus, the Intuitive Method has a possibility of 0.5 to fail at the second step. Even though the algorithm, fortunately, picked *h* or *j* at the second step, it is still likely to fail at the following steps. When faced with pentagrams, the Intuitive Method only needs five steps to establish the whole mapping, because each step is a successful trial. However, it is difficult for its speedy answer to be correct.

### 4.2. Results of IsoMarking

**Figure 3:**
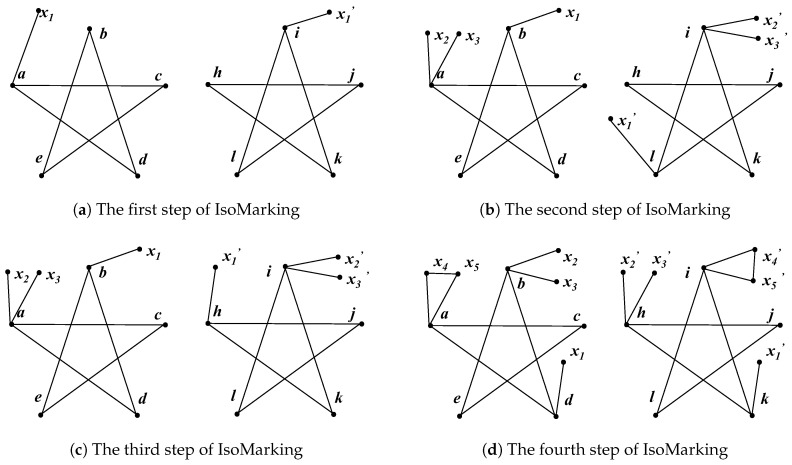
Each step of IsoMarking running on pentagrams. (**a**) The algorithm chose *a* and *i*. The generated graphs are the same, and thus, a→i was established. (**b**) The algorithm chose *b* and *l*. With *a* and *i* marked, the new graphs were non-isomorphic and therefore, the bijection b→l failed. (**c**) The algorithm chose *b* and *h*, and thus established b→h. (**d**) The algorithm chose *d* and *k*, and then established d→k. Note that the marked graphs are no more symmetric after the third step.

As for IsoMarking, the consequence becomes different. Since there is no existing bijection at the first step, IsoMarking only marks the nodes of the current candidate bijection. It randomly picks one node from each graph and marks them, similar to the Intuitive Method. We assumed that it also chose *a* and *i*, and thus, the new graphs shown in Figure 3a were generated. Obviously, these graphs are the same and IsoMarking accepted a→i.

Suppose that IsoMarking also picks *b* and *l* at the second step, and then, it marks *b* and *l* using the first marking type and marks *a* and *i* using the second type. Obviously, the new graphs (shown in Figure 3b) are no more isomorphic; therefore, the bijection b→l fails. Because nodes of the previous bijection are marked, the symmetry is strongly reduced. We can see that marking vertices does help IsoMarking to reject b→l which conflicts with a→i.

At the third step, IsoMarking has to test some other unit bijection candidates. As long as IsoMarking chooses node *b* in the left, only *h* and *j* in the right are qualified. We assumed that IsoMarking picked node *h* at the third step, and then new graphs were generated, as shown in Figure 3c. These two graphs are isomorphic, and thus b→h is considered to beconsistent with a→i. As a result, IsoMarking has two unit bijections {a→i,b→h}.

At the fourth step, IsoMarking continues marking two existing bijections {a→i,b→h}. With two existing bijections marked, the graphs are no longer symmetric. Hence, they become ordinary graphs, and the other unit bijections can be easily revealed: {c→l,e→j,d→k}. We assumed IsoMarking chose *d* and *k*, and the new graphs are shown in Figure 3d. With isomorphic graphs, IsoMarking establishes d→k. By marking existing bijections, IsoMarking can accept those unit bijections one by one and work out a correct isomorphism mapping eventually.

Consequently, we can see the symmetry of pentagrams is strongly reduced, and unit bijections established by trials will never be in conflict with each other. The pentagram is highly symmetric, and all its nodes are equivalent to each other. However, after the third step of IsoMarking, the marking of two existing bijections is sufficient to change pentagrams into ordinary graphs. Therefore, different marking types can collaborate and become even more powerful. As a result, IsoMarking only needs, in total, three marking types when faced with pentagrams. Although IsoMarking needs more steps due to failed trials, it can obtain correct mapping eventually. In the case of pentagrams, IsoMarking can always find a correct answer while the Intuitive Method is more likely to fail.

## 5. Experiments

In this section, we conduct experiments to evaluate IsoMarking. Firstly, we introduce information concerning the experimental setup. Then, we compare our algorithm with state-of-the-art algorithms on both ordinary graphs and regular graphs. Finally, we study the running costs of these algorithms.

### 5.1. Experimental Setup

We aimed to choose state-of-the-art algorithms to compare the performance. As discussed in Section 1, some of the most influential ones, including VF2 [12], VF3 [13,14], RI [5], and LAD [15], are usually based on the tree search and verification. These algorithms are optimal with perfectly correct results. Nevertheless, the complexity is exponential or quasi-polynomial in the worst cases. IsoMarking, in contrast, is based on random walks. It is similar to approximation algorithms. IsoMarking is polynomial-time in any graph, although the accuracy is relatively limited. Therefore, it is difficult to compare IsoMarking with optimal algorithms for accuracy, because the optimal algorithms are always correct. Likewise, it is difficult to compare them in terms of complexity, because IsoMarking is theoretically superior in computational complexity. Such a comparison does both sides an injustice.

Therefore, we only compared IsoMarking with other algorithms based on random walks. For better comparison, we chose polynomial-time algorithms with sacrificed accuracy. Thus we chose the Intuitive Method [24], Emms [21], and Qiang3 [24] for comparison. The Intuitive Method can discover an isomorphism mapping based on a judging algorithm (Qiang1) [23]. Emms [21,22] is most influential in the graph isomorphism problem, and we chose the continuous-time version [21]. We also chose Qiang3 [24] as a baseline, as it is claimed to perform particularly well on regular graphs. All the source codes were implemented in Matlab. Some source codes are available from the thesis [24], and we implemented the other codes, including an implementation of Emms according to its related works [21,36].

Because those algorithms are not perfectly correct, we concentrated on the accuracy performance. We evaluated the algorithms with two experiments. The first experiment focused on their performance on ordinary graphs, while the second was designed to evaluate them on regular graphs.

The two experiments were carried out in the same way except for the graph type. Firstly we generated groups of graph paisr. Each pair included two isomorphic graphs. We aimed to evaluate their accuracy of discovering an isomorphism mapping; thus, we performed algorithms on every graph pair and examined the output mapping. There can be more than one correct isomorphism mapping, especially in regular graphs. Therefore, it is not suitable to compare the output mapping with the ground truth. We returned to the definition of isomorphism mapping in Definition 1. Thus, we chose to check whether the output mapping was structure-preserving instead. In our experiments, we depended on Algorithm 3 [24] to verify the output. If the mapping passed the verification, and then it was deemed to be correct; otherwise, the mapping was deemed to have failed. The average accuracy of each algorithm in every group was calculated. Furthermore, we recorded the average running time and the peak memory cost while running each algorithm based on the Profiler in Matlab.

**Algorithm 3** Check
1:**Input: **$G_{1}=\left(V_{1},E_{1}\right),G_{2}=\left(V_{2},E_{2}\right),$ and the mapping *f*;2:
**if** $\exists v_{1}\in V_{1},\forall v_{2}\in V_{2},\left\{v_{1}\to v_{2}\right\}\notin f$ **then**
3:    Pass=False;4:    **return** Pass;5:
**end if**
6:**for** each $v_{i}\in V_{1}$ **do**7:    **for** each $v_{j}\in V_{1}$ **do**8:        **if** $\left(\left(\left\{v_{i},v_{j}\right\}\in E_{1}\right)\neq \left(\left\{f\left(v_{i}\right),f\left(v_{j}\right)\right\}\in E_{2}\right)\right)$ **then**9:           Pass=False;10:           **return** Pass;11:        **end if**12:    **end for**13:
**end for**
14:Pass=True;15:**Output: **the result Pass.


We generated 16 groups of graphs with 1585 pairs of isomorphic graphs in total. All experiments were performed on a 2.6 GHz Intel i5-4210M PC with 8 GB main memory, running Windows 8. Matlab codes were run in 64-bit Matlab R2012b (8.0.0.783).

### 5.2. Results on Ordinary Graphs

We generated six groups of ordinary graphs, namely Groups 1–6. Each group consisted of 100 ordinary graph pairs. Hence, there were 600 pairs of ordinary graphs in total. The basic information about Groups 1–6 is presented in Table 3, where *N* and *k* refer to the vertex number and the average degree, respectively.

**Figure 4:**
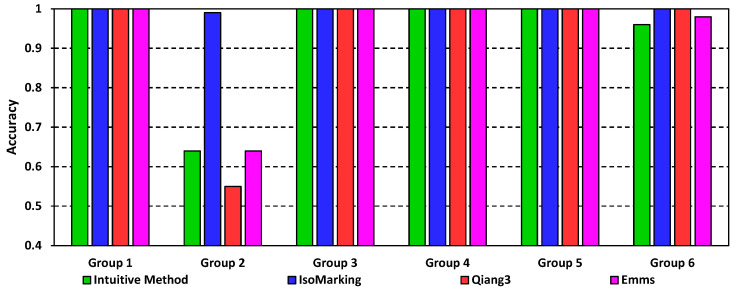
Accuracy results on ordinary graphs.

The results are shown in Figure 4. All algorithms performed well in most groups. In Group 1 and Groups 3–5, all of the algorithms performed perfectly with an accuracy of 1.00. In Group 6, the Intuitive Method and Emms achieved 0.98 and 0.96, respectively. They were only a little worse than IsoMarking and Qiang3 whose accuracies were both 1.00. Group 2 was relatively distinctive. Because Group 2 had larger and more complicated graphs, it was more challenging. Therefore, various algorithms performed differently, and thus, the performance of some algorithms greatly dropped. Even so, IsoMarking still performed the best with a score of 0.99. On the contrary, the figures of the other algorithms were only about 0.55 or 0.64.

There was another interesting phenomenon. In the 600 graph pairs, IsoMarking rarely performed worse than any other algorithm. The only exception was the nineteenth graph pair in Group 2, where only Qiang3 gave the correct isomorphism mapping. For the other 599 graph pairs, IsoMarking definitely worked out a correct mapping, while the other algorithms failed occasionally.

As for the promotion of IsoMarking, we only studied the promotion in Group 2, because the figures for the other groups were very close. In the second group, IsoMarking outperformed the Intuitive Method and Emms by 54.69%, and it outperformed Qiang3 by 80%.

So, in ordinary graphs, we can see that IsoMarking performs the best. Although the others usually perform well, their performance drops greatly when the graphs become complicated. IsoMarking not only keeps an accuracy level that is never less than 0.99, but it also outperforms others considerably when graphs become more challenging.

### 5.3. Results on Regular Graphs

We also generated more regular graphs for experiments. There were 985 pairs of regular graphs in total, divided into ten groups, namely Groups 7–16. The basic information about these groups is shown in Table 4.

The results for regular graphs are shown in Table 5. Obviously, various algorithms performed significantly differently.

Emms had the worst performance with an accuracy level that was usually close to 0. The only exception was Group 15, in which its accuracy was 0.5. The Intuitive Method was also unsatisfying, although it was much better than Emms. Its score was always less than 0.5, except for Group 7. Its accuracy was even less than 0.3 in six groups. These two algorithms lack effective techniques to cope with the impact of symmetry. Therefore, they are easily confused by regular graphs and often fail to give a correct isomorphism mapping.

Our experiments demonstrated that Qiang3 achieves better performance on regular graphs. Its accuracy was usually about 0.5 to 0.6, and even 0.8 sometimes. In Group 11, Qiang3 even achieved a score very close to that of IsoMarking. Consequently, Qiang3 performed much better than the other two baselines in regular graphs, although its performance on ordinary graphs was relatively low. Qiang3 scored zero in Group 15, but such a case can be regarded as an exception considering its overall accuracy. Qiang3 was able to reduce the impact of symmetry to some extent and utilized verification to avoid conflicts between unit bijections. Therefore, it usually performed better than the Intuitive Method and Emms.

IsoMarking performed best in all groups. Its accuracy was usually higher than 0.75. It was the only algorithm whose accuracy reached 0.85. It scored 0.85 or higher in six groups. Even in Group 15, where two algorithms were utterly wrong, its accuracy was still not less than 0.75. With its superior performance, IsoMarking achieved brilliant promotion. It outperformed the Intuitive Method by 43.75–466.67%, and it outperformed Qiang3 by 3.61–48.72%, not to mention Emms whose score was usually 0.

When we analysed the results from the perspective of groups, we drew similar conclusions. In all ten groups, IsoMarking was always the best. Qiang3 usually performed well, followed by the Intuitive Method. Emms usually scored close to zero. There was an exception (Group 15), where Emms was superior to other baselines, but IsoMarking still performed the best.

For the 985 pairs of regular graphs, IsoMarking never performsedworse than the Intuitive Method or Emms. In other words, when IsoMarking failed to work out a correct isomorphism mapping between two regular graphs, only Qiang3 could still give a correct mapping between them. However, such cases were rare—there were about only 50 cases in the total 985 cases (5.076%). In such cases, the four marking types were not sufficient, thus IsoMarking required more marking types to work out a correct mapping. In the other cases, if IsoMarking could not work out a correct mapping, then no algorithm was able to do so.

In this subsection, the Intuitive Method and Emms were shown to have very limited performance in regular graphs, and the performance of Qiang3 was not always satisfying, either. IsoMarking, however, still maintained its good performance. We can safely draw a conclusion that the mechanism of marking vertices does have the ability to reduce the impact of symmetry which helps IsoMarking to perform much better on regular graphs.

### 5.4. Running Cost Study

In this subsection, we describe the study of the running cost, including the time cost and the memory cost. Since all codes were implemented in Matlab, we used the profiler in Matlab to record the cost. The peak memory in the profile summary was chosen as the peak memory cost of each algorithm, as shown in Table 6. As for the time cost, the verification time spent on Algorithm 3 was not included. The average running time of each algorithm in each group was calculated, as shown in Table 7.

Generally, the running cost is related to the algorithm, the group size and the graph size. Each group is processed in a batch. Thus, more memory is required if the group has more graph pairs, but the average running time is not influenced. When faced with larger graphs, algorithms usually require more memory and time, which is particularly obvious with Emms. In the following discussion, we mainly discuss the running costs of different algorithms.

The results of the memory cost were very different among groups. However, we can still obtain some conclusions. Emms is the most memory-consuming because it used the largest amount of memory in none groups. Emms conducts a quantum walk on a graph much larger than the original graphs, and thus, it definitely requires more memory. Moreover, when the graphs became larger in Group 2 and Group 7, the figure of Emms increased significantly. Likewise, Qiang3 is memory-consuming, although it is a little better than Emms. Its memory cost is the largest in four groups. The Intuitive Method performed better than Emms and Qiang3, but IsoMarking performed the best. The figure of IsoMarking was the least with six groups, while it used the largest amount of memory only in Group 9. As a consequence, the memory cost of IsoMarking was satisfying.

As for time cost, both Qiang3 and Emms performed the quantum walk only once, thus they are faster. Qiang3 was usually faster in ordinary graphs, while Emms was faster in regular graphs. In regular graphs, however, Emms simply considers all unit bijections to be acceptable. It returns a mapping almost immediately, regardless of the fact that the mapping can be incorrect. As a consequence, Emms usually scores zero in regular graphs, and thus such speed is meaningless. Therefore, Qiang3 performs the best. In fact, Qiang3 is computationally faster, which is $O(|V{|}^{4})$.

IsoMarking and the Intuitive Method, in contrast, take more time. IsoMarking is even slower. Both algorithms are $O(|V{|}^{6})$, and they require repeated quantum walks to establish unit bijections one after another. Thus, they spend a large amount of time conducting quantum walks. The Intuitive Method can accept unqualified unit bijections, and return a mapping more quickly, despite the fact that the mapping can be incorrect. IsoMarking, however, utilizes different marking types, thus it is more strict. It rejects unqualified unit bijections accepted by the Intuitive Method, and therefore, it takes more trials, as discussed in Section 4. Furthermore, IsoMarking adds vertices to graphs. Although those nodes have little influence on the theoretical complexity, they can increase the time cost to some extent.

From what has been discussed above, we can see that IsoMarking performs well on memory cost, but it is somewhat time-consuming. Therefore, the overall running cost is barely satisfactory. Considering its brilliant promotion on both ordinary graphs and regular graphs, we believe such cost is acceptable.

## 6. Conclusions

We propose IsoMarking to figure out the isomorphism mapping between graphs. It uses the continuous-time quantum walk to sense topological changes in graphs. By marking vertices, it strongly reduces the symmetry and keeps bijections consistent with each other. The experiments in this study demonstrated that IsoMarking rarely performs worse than other algorithms. It achieved higher accuracy when discovering isomorphism mappings. Moreover, it significantly outperformed other algorithms when graphs were highly symmetric, especially for regular graphs. Its running cost was also acceptable. We plan to optimize our algorithm so that it can be more efficient and effective. Likewise, we plan to study the marking mechanism more deeply, so that it can cope with some extremely difficult structures.

## Figures and Tables

**Table 1:** Continuous-time quantum walk (CTQW) on the left pentagram.

Node	*t* = 1.0	*t* = 2.0	*t* = 3.0
*a*	0.3251 − 0.0740*i*	0.3747 − 0.1674*i*	0.3284 + 0.2593*i*
*b*	0.3251 − 0.0740*i*	0.3747 − 0.1674*i*	0.3284 + 0.2593*i*
*c*	0.3251 − 0.0740*i*	0.3747 − 0.1674*i*	0.3284 + 0.2593*i*
*d*	0.3251 − 0.0740*i*	0.3747 − 0.1674*i*	0.3284 + 0.2593*i*
*e*	0.3251 − 0.0740*i*	0.3747 − 0.1674*i*	0.3284 + 0.2593*i*

**Table 2:** CTQW on the right pentagram.

Node	*t* = 1.0	*t* = 2.0	*t* = 3.0
*h*	0.3251 − 0.0740*i*	0.3747 − 0.1674*i*	0.3284 + 0.2593*i*
*i*	0.3251 − 0.0740*i*	0.3747 − 0.1674*i*	0.3284 + 0.2593*i*
*j*	0.3251 − 0.0740*i*	0.3747 − 0.1674*i*	0.3284 + 0.2593*i*
*k*	0.3251 − 0.0740*i*	0.3747 − 0.1674*i*	0.3284 + 0.2593*i*
*l*	0.3251 − 0.0740*i*	0.3747 − 0.1674*i*	0.3284 + 0.2593*i*

**Table 3:** Information about the ordinary graph groups.

Group Name	# of Graph Pairs	*N*	Average *k*
Group 1	100	17	10.2353
Group 2	100	34	4.5294
Group 3	100	18	3
Group 4	100	18	4.1111
Group 5	100	20	3.8
Group 6	100	10	3

**Table 4:** Information about the regular graph groups.

Group Name	# of Graph Pairs	*N*	*k*
Group 7	100	30	3
Group 8	149	16	3
Group 9	100	14	4
Group 10	200	14	3
Group 11	100	11	6
Group 12	100	11	4
Group 13	85	12	3
Group 14	60	10	5
Group 15	32	20	3
Group 16	59	10	4

**Table 5:** Accuracy results for regular graphs.

Group Name	Intuitive Method	IsoMarking	Qiang3	Emms
Group 7	0.64	0.92	0.64	0
Group 8	0.3758	0.8993	0.6846	0
Group 9	0.28	0.68	0.56	0
Group 10	0.28	0.87	0.585	0
Group 11	0.44	0.86	0.83	0
Group 12	0.4	0.9	0.83	0.02
Group 13	0.1529	0.8353	0.5765	0.0118
Group 14	0.15	0.85	0.6167	0
Group 15	0	0.75	0	0.5
Group 16	0.2203	0.7458	0.6102	0

**Table 6:** Results of the peak memory cost.

Group Name	Intuitive Method	IsoMarking	Qiang3	Emms
Group 1	3.8203 MB	3.6328 MB	2.8750 MB	3.9648 MB
Group 2	6.3867 MB	4.6367 MB	5.5000 MB	39.9141 MB
Group 3	5.5078 MB	4.6875 MB	6.8242 MB	7.2969 MB
Group 4	4.9492 MB	5.8750 MB	2.7578 MB	9.6641 MB
Group 5	4.5000 MB	3.0664 MB	4.4453 MB	4.4531 MB
Group 6	3.5000 MB	3.8203 MB	5.2500 MB	3.6328 MB
Group 7	5.6367 MB	6.1367 MB	5.8125 MB	21.1484 MB
Group 8	6.3750 MB	1.5000 MB	1.7930 MB	3.2461 MB
Group 9	4.0000 MB	7.0000 MB	5.5625 MB	4.6328 MB
Group 10	3.0664 MB	3.3203 MB	3.5000 MB	2.9258 MB
Group 11	0.5938 MB	0.6875 MB	0.7500 MB	1.0625 MB
Group 12	4.1992 MB	3.5000 MB	4.3125 MB	6.8281 MB
Group 13	0.1250 MB	0.1484 MB	0.1523 MB	0.3164 MB
Group 14	1.8750 MB	3.7539 MB	4.5000 MB	0.5039 MB
Group 15	3.3750 MB	3.3125 MB	3.2500 MB	6.8867 MB
Group 16	3.2500 MB	2.0000 MB	3.6250 MB	0.5000 MB

**Table 7:** Results of the average running time.

Group Name	Intuitive Method	IsoMarking	Qiang3	Emms
Group 1	3.2654 s	3.2171 s	0.0108 s	0.0969 s
Group 2	44.8457 s	54.0828 s	0.8583 s	4.5173 s
Group 3	3.7842 s	5.1633 s	0.0141 s	0.1277 s
Group 4	3.6923 s	5.9058 s	0.0141 s	0.1221 s
Group 5	5.3259 s	7.8721 s	0.0162 s	0.2217 s
Group 6	0.5962 s	0.9774 s	0.0062 s	0.0106 s
Group 7	22.5406 s	30.4999 s	0.3093 s	1.9541 s
Group 8	3.1396 s	3.7451 s	0.1894 s	0.1843 s
Group 9	2.0971 s	2.5978 s	0.2170 s	0.0489 s
Group 10	2.0007 s	2.5475 s	0.2040 s	0.0501 s
Group 11	0.9494 s	1.2719 s	0.0892 s	0.0278 s
Group 12	1.0031 s	1.2552 s	0.1000 s	0.0232 s
Group 13	1.3539 s	1.5960 s	0.1858 s	0.0260 s
Group 14	1.0457 s	0.9848 s	0.1669 s	0.0181 s
Group 15	6.7206 s	7.7323 s	0.5846 s	0.2707 s
Group 16	1.1468 s	1.1151 s	0.1750 s	0.0204 s

## Abbreviations

The following abbreviations are used in this manuscript:

LAD	Local All Different
CTQW	Continuous-time quantum walk
DTQW	Discrete-time quantum walk
NP	Nondeterministic Polynomial-time
